# Why We Shouldn’t Ask PACU Patients: ‘Are You Having Pain?’

**Published:** 2022-12-22

**Authors:** Barry L Friedberg, Andrew Mannes

**Affiliations:** 1 Goldilocks Anesthesia Foundation, CA 92658, USA; 2 Department of Perioperative Medicine, Clinical Center, NIH, MD 20892, USA

In 1999, Macario, et al. published two scientifically validated surveys, one for anesthesiologists and a separate one for patients [1,2]. Both groups were asked to rank in order of importance the *most important* postoperative outcomes to avoid. Anesthesiologists ranked pain as ^#^1. Surprisingly, patients ranked emesis ^#^1, gagging on the endotracheal tube ^#^2, and nausea ^#^3. Patients ranked pain as a distant ^#^4 outcome to avoid! Since anesthesiologists, as well as Post Anesthesia Care Unit (PACU) Registered Nurses, are laser focused on pain, why do patients rank emesis well ahead of pain?

Violation of the integument is a common experience often accompanied with pain. Emesis is not usually associated with a cut in the integument. Patients’ outcomes ^#^1 and ^#^3 are the reason we group those experiences as postoperative nausea *and* vomiting (PONV). When patients sign a surgical consent, they are giving their surgeons permission violate their integument with a sharp scalpel or a trocar. *Pain is the natural expectation of integument violation.* Asking PACU patients a closed end question like, ‘Are you having pain?’ plays into their pre-surgical expectation.

High anxiety patient populations like Eastern European derived (Ashkenazi) Jewish, Hispanic and Arabic ones have heightened expectations of surgical pain. Pain complaints are likely from culturally acquired expectations rather than from their somatic experience. In PACU, these patients may benefit from acetaminophen 1,000 mg plus diphenhydramine 50 mg therapy, either intravenously or orally (i.e., Tylenol PM^™^) instead of additional opioids. To the occidental anesthesiologist, Asian patients appear more stoic. These patients more commonly view pain as a natural part of life and are less apt to complain.

In 1999, in an Apfel-defined high PONV risk population (i.e., nonsmoking females with PONV history having emetogenic cosmetic surgery with propofol ketamine anesthesia), a 0.6% PONV rate without antiemetics was published [3]. This 0.6% rate was favorably compared to the 7% PONV rate reported by White, et al. in Apfel’s PONV chapters in Miller’s Anesthesia [4,5]. In both chapters, Apfel also remarked, “As long as emetogenic agents are part of the anesthetic regimen, the use of antiemetics is of limited utility.” Apfel’s admonition was ignored in a recent consensus guideline paper that continued to recommend antiemetics [6]. The absence of a dedicated PONV chapter in the 2020 edition of Miller’s Anesthesia textbook does not say much for our profession’s regard for our patients’ number one concern. Emesis may not often happen on the OR table. It is no less distressing to our patients.

The long held anesthesia syllogism is ‘Surgery is painful, opioids are pain killers, therefore, all surgery requires the (judicious) use of opioids.’ Opioid Free Anesthesia or why it’s never easy to go against long established practice [7]. Trying to eliminate surgical pain with opioids is akin to trying to extinguish a fire with gasoline [8].

Pain and PONV remain the two most common causes for unexpected admission after outpatient surgery. Over a two decades in more than 4,000 *opioid free*, cosmetic surgery outpatients, not a single pain or PONV admission despite all patients having had commercial insurance to cover such an eventuality [9]. Some office based recovery personnel more constructively ask patients, ‘Are you comfortable’ instead of asking ‘Do you have pain?’ Whether such a verbal effort proves to change pain outcomes and reduce pain medication usage needs to be investigated using larger datasets or possibly clinical trials. Based on clinical observation, there appear to be good reasons to *not* ask PACU patients if they have pain, a both objective and subjective phenomenon. It is probable that to drawing patient’s attention to pain could increase pain related complaints and possibly pain medication requirement, including opioid administration. The institution of pain as a vital sign and publications implying improved postoperative courses with pain reduction could have driven clinicians to be obsessed with trying to eliminate pain by asking patients if they had pain. Subsequent administration of opioids could result in more PONV. The overuse of postoperative opioid use has led to addiction and a modified immune system [10,11].
